# Effects of Progressive Replacement of Corn Stover with Condensed Tannin-Containing Woody Biomass on Growth Performance and Reproductive Development of Prospective Breeding Ram Lambs

**DOI:** 10.3390/ani16203161

**Published:** 2026-10-09

**Authors:** Octavio Carrillo-Muro, Alejandro Rivera-Villegas, Pedro Hernández-Briano, Carlos Aurelio Medina-Flores, Marco Antonio López-Carlos, Carlos Fernando Aréchiga-Flores, Francisco J. Gutiérrez-Piña, Oliver Yaotzin Sánchez-Barbosa, Daniel Rodríguez-Cordero, Alejandro Plascencia

**Affiliations:** 1 Unidad Académica de Medicina Veterinaria y Zootecnia, Universidad Autónoma de Zacatecas, General Enrique Estrada 98500, Zacatecas, Mexico; octavio_cm@uaz.edu.mx (O.C.-M.); alejandro.rivera@uaz.edu.mx (A.R.-V.); carlosmedina@uaz.edu.mx (C.A.M.-F.); lopcarmarco@uaz.edu.mx (M.A.L.-C.); arechiga@uaz.edu.mx (C.F.A.-F.); fpina@uaz.edu.mx (F.J.G.-P.); oliver.sanchez@uaz.edu.mx (O.Y.S.-B.); 2 Instituto de Investigaciones en Ciencias Veterinarias, Universidad Autónoma de Baja California, Mexicali 21100, Baja California, Mexico; daniel.rodriguez64@uabc.edu.mx; 3 Facultad de Medicina Veterinaria y Zootecnia, Universidad Autónoma de Sinaloa, Culiacan 80260, Sinaloa, Mexico; alejandro.plascencia@uabc.edu.mx

**Keywords:** dietary palatability, feed efficiency, income over feed cost, *Quercus rugosa*, semen quality, sustainable feeding

## Abstract

This study determined the level at which corn stover (CS) could be progressively replaced with condensed tannin-containing woody biomass (CTWB) from *Quercus rugosa* without compromising dietary acceptance, growth, energy utilization, or reproductive development in prospective breeding ram lambs. In Experiment 1, eight intact Rambouillet ram lambs (5 months old; 40.7 ± 0.2 kg initial body weight) were simultaneously offered diets containing 0–80% CTWB (CTWB-0–CTWB-80), equivalent to 0.00–20.96 g condensed tannins (CT)/kg dry matter (DM), in an 8-d preference trial. In Experiment 2, 36 intact Rambouillet ram lambs (6 months old; 49.97 ± 0.79 kg initial body weight) were assigned for 70 d to diets containing 0–60% CTWB and a calculated CT contribution from CTWB of 0.00–15.72 g/kg DM (*n* = 9/treatment). In the preference trial, CTWB-20 reduced CS from 80 to 60% without affecting the proportion of diet consumed or relative palatability index compared with CTWB-0, whereas inclusions ≥40% reduced both responses (*p* = 0.0143 and *p* = 0.0146, respectively). In the growth trial, CTWB-20, corresponding to 5.24 g CT/kg DM from CTWB, maintained growth performance comparable with CTWB-0 and produced a greater overall growth response than CTWB-60, reflected by greater final body weight (63.95 vs. 57.87 kg; *p* = 0.016), average daily gain (0.202 vs. 0.126 kg/d), cumulative body weight change (14.19 vs. 8.93 kg; *p* = 0.019), and gain-to-feed ratio (0.094 vs. 0.058; *p* = 0.011). Dry matter intake (DMI) remained similar (*p* = 0.278). As CTWB increased, expected DMI increased (*p* < 0.0001), the observed-to-expected DMI ratio decreased (*p* = 0.003), and the observed-to-expected net energy for maintenance (NE_m_) and gain (NE_g_) ratios increased (*p* ≤ 0.045), although observed NE_m_ and NE_g_ concentrations remained unchanged (*p* = 0.146). Rib fat thickness was maintained with CTWB-20 but decreased with CTWB-40 and CTWB-60 (*p* = 0.004), whereas longissimus muscle area, scrotal circumference, and sperm motility remained similar (*p* ≥ 0.157). Additionally, CTWB-20 was associated with greater sperm viability (*p* = 0.032), while all CTWB levels were associated with fewer morphologically abnormal sperm (*p* = 0.005). Finally, treatment did not affect income over feed cost or cost of gain (*p* = 0.217 and *p* = 0.735, respectively), indicating that partial replacement with CTWB did not adversely affect economic efficiency. Overall, 20% CTWB reduced CS from 60 to 40% and provided a favorable balance among dietary acceptance, growth, semen characteristics, and economic efficiency.

## Simple Summary

In extensive production systems, forage availability declines during the dry season, creating a need for locally available fiber sources that can complement or partially replace conventional feed resources. Condensed tannin-containing woody biomass (CTWB) from *Quercus rugosa* accumulates during this period and provides both fiber and condensed tannins, which may influence nutrient utilization and reproductive responses. Preference and growth trials were conducted to evaluate the progressive replacement of corn stover (CS) with CTWB in prospective breeding ram lambs. Including 20% CTWB partially replaced CS without reducing dietary acceptance in the preference trial or feed intake during the growth trial. In the latter, this inclusion reduced CS from 60 to 40% of the diet and maintained growth performance comparable with CTWB-0 while supporting greater growth and feed efficiency than complete CS replacement with CTWB-60. It also maintained observed dietary energy, subcutaneous fat, muscle development, scrotal growth, and economic efficiency while being associated with greater sperm viability and fewer sperm abnormalities. Conversely, higher inclusion levels reduced palatability and did not maintain the productive response observed with 20% CTWB. Therefore, including 20% CTWB may provide a practical strategy to reduce CS use in diets for prospective breeding rams during the dry season.

## 1. Introduction

In forage-based extensive ruminant production systems, pastures and crop residues provide the fibrous base and account for the largest proportion of dietary dry matter (DM) [1,2]. However, their availability and quality decline during the dry season, creating a seasonal nutritional gap across different ruminant production systems [3]. Among these resources, corn stover (CS) is primarily available after harvest, but its supply decreases as the dry season progresses. When on-farm production is insufficient, some producers must purchase CS or obtain forage from external sources, thereby increasing feeding costs [1]. This limitation is particularly relevant for prospective breeding ram lambs, whose diets must simultaneously support body growth, testicular development, and future reproductive capacity [4,5,6]. Consequently, locally available fiber sources that seasonally complement conventional forages are needed.

In this context, trees and shrubs can provide biomass when herbaceous vegetation becomes scarce [7]. These resources include condensed tannin-containing woody biomass (CTWB), which supplies fiber and nutrients as well as biologically active condensed tannins (CT). In certain forage species, concentrations of 20–45 g CT/kg DM may reduce ruminal crude protein (CP) degradation, increase the intestinal flow of amino acids, and contribute to the control of bloat and gastrointestinal parasites [8,9]. Plant materials rich in CT and other phenolic compounds may also improve testicular antioxidant capacity and some sperm characteristics [10]. In prepubertal lambs, grape seed tannin extract supplying 2.03 and 4.07 g CT/kg DM improved antioxidant capacity and some indicators of testicular development without affecting dry matter intake (DMI), growth, or scrotal circumference (SC) [11]. Therefore, CTWB could contribute to the nutrition and reproductive development of young males, although its effects depend on the origin, structure, and reactivity of CT [12].

Among these resources, species of the genus *Quercus* are widely distributed throughout the Northern Hemisphere, and their foliage is used as feed for small ruminants in different regions [13]. In India and Nepal, some species are offered fresh or stored for periods of forage scarcity [14], whereas in the United States, *Q. wislizenii* biomass has been evaluated as sheep feed [15]. In Mexico, where the genus is highly diverse [16], the natural defoliation of *Q. rugosa* occurs from March to April, coinciding with the dry season. Condensed tannin-containing woody biomass accumulated during this period contains approximately 22.3–26.2 g CT/kg DM, and its accumulation can reach 616 kg DM/ha during the first 15 d of collection [17]. This biomass can also replace up to 30% of CS in maintenance diets without reducing palatability. Similarly, in a preference trial, CTWB from *Q. resinosa* completely replaced the forage component of diets formulated for finishing lambs without reducing intake relative to a diet without CTWB [17,18]. Therefore, *Quercus* CTWB may complement conventional forage supplies during periods of scarcity while providing CT with potential nutritional and biological effects.

Nevertheless, the proportion at which *Q. rugosa* CTWB can replace CS without compromising dietary acceptance, growth, and reproductive development in prospective breeding ram lambs remains unknown, as do the energy and economic consequences of the concomitant increase in CT. Therefore, this study aimed to determine the appropriate level of progressive CS replacement with *Q. rugosa* CTWB and evaluate its effects on intake and palatability, growth performance, feed efficiency, energy utilization, ultrasound measurements, scrotal development, semen characteristics, and economic outcomes in Rambouillet lambs selected as prospective breeding rams. We hypothesized that a moderate inclusion of CTWB would reduce CS use without compromising dietary acceptance, growth, or reproductive development while improving economic returns.

## 2. Materials and Methods

### 2.1. Study Site, Ethical Approval, and General Procedures

The intake and palatability trial was conducted in June 2013, whereas the growth performance trial was conducted from January to May 2014 at the Small Ruminant Performance Testing Facility of the Unidad Académica de Medicina Veterinaria y Zootecnia, Universidad Autónoma de Zacatecas (UAMVZ-UAZ), located in north-central Mexico (22°58′49.09″ N, 102°42′57.26″ W; 2157 m above sea level). The chemical composition and in vitro gas production data for CTWB were obtained from the same collection previously characterized at the Animal Nutrition Laboratory and reported by Carrillo-Muro et al. [17]. Corn stover was analyzed at the Animal Nutrition Laboratory, whereas semen evaluations were conducted at the Animal Reproduction Laboratory; both laboratories belong to UAMVZ-UAZ. The lambs used in both experiments were privately owned and were provided by their owner for the purposes of this study.

Animal handling and transportation complied with Mexican regulations [19,20,21]. The experimental protocol was reviewed and approved by the Institutional Committee on Bioethics and Animal Welfare of UAMVZ-UAZ before the study began (Protocol No. 2013/05/19; Date of Approval: 19 May 2013). During both experiments, trained personnel monitored the animals daily to assess their health, feed intake, and general welfare.

At enrollment, none of the lambs showed evident clinical signs of disease. No additional inclusion or exclusion criteria were prespecified beyond the animal characteristics described for each experiment. A priori humane endpoints for removal included marked body weight loss, inability to eat or drink, recumbency, severe disease, digestive disorders, or locomotor abnormalities that compromised animal welfare. No animals reached these endpoints or experienced adverse events requiring removal; therefore, all animals completed their respective experiments and were included in the analyses.

### 2.2. Condensed Tannin-Containing Woody Biomass Collection, Processing, and Chemical Composition

The *Q. rugosa* CTWB used in both experiments came from the collection characterized by Carrillo-Muro et al. [17]. The material was collected from 1 March to 30 April 2013, during the dry season and natural defoliation, at Rancho Arroyo de Carrillo in the municipality of Tequila, Jalisco, Mexico (21°15′07.30″ N, 103°39′48.12″ W; 1617 m above sea level). It was ground in a hammer mill through a 25-mm screen and stored in laminated bags on pallets in a dry facility protected from light, moisture, and pests. In that study, representative CTWB samples were analyzed in triplicate. Dry matter (DM) and ash were determined according to AOAC [22]; crude protein (CP) was quantified by Dumas combustion using an FP-528 nitrogen analyzer (LECO Corporation, St. Joseph, MI, USA); ether extract (EE) was determined with an ANKOM^XT15^ extractor (ANKOM Technology, Macedon, NY, USA) according to official method Am 5-04 [23]; and neutral detergent fiber (NDF) was determined without α-amylase according to Van Soest et al. [24]. Condensed tannins were quantified using the butanol–HCl assay described by Porter et al. [25]. The stored CTWB was not reanalyzed immediately before Experiment 2; therefore, diet formulation used the chemical composition and CT concentration determined for the collected material.

In the same characterization, in vitro gas production was measured in quadruplicate at 24 h of incubation according to Theodorou et al. [26], and metabolizable energy (ME) was estimated from gas production and chemical composition according to Menke and Steingass [27]. For the present diet calculations, digestible energy (DE) was calculated as DE = ME/0.82 and total digestible nutrients (TDN) as TDN (%) = DE/0.04409. Net energy for maintenance (NE_m_) and net energy for gain (NE_g_) were estimated as NE_m_ = 1.37(ME) − 0.138(ME^2^) + 0.0105(ME^3^) − 1.12 and NE_g_ = 1.42(ME) − 0.174(ME^2^) + 0.0122(ME^3^) − 1.65, respectively [28], using the unrounded ME value. Based on the characterization reported by Carrillo-Muro et al. [17], CTWB contained 85.0% DM and, on a DM basis, 3.9% CP, 1.5% EE, 58.2% NDF, 6.0% ash, and 2.62% CT. Its unrounded ME estimate was 1.522 Mcal/kg DM; the rounded values used for diet formulation were 1.5 Mcal ME/kg DM, 42.1% TDN, 0.7 Mcal NE_m_/kg DM, and 0.2 Mcal NE_g_/kg DM.

Corn stover was ground through a 25-mm screen to obtain a particle size comparable to that of CTWB. Representative CS samples were analyzed in triplicate using the corresponding chemical procedures described above. Corn stover contained 89.0% DM and, on a DM basis, 6.5% CP, 2.1% EE, 65.0% NDF, and 7.2% ash. Its energy contribution was based on the NRC [5] tabular values for “Corn Stalks, Grazing”: 2.4 Mcal ME/kg DM, 65.9% TDN, 1.5 Mcal NEm/kg DM, and 0.9 Mcal NEg/kg DM. Condensed tannins were not determined in CS; therefore, the reported dietary CT values represent only the calculated contribution from CTWB, based on its analyzed CT concentration and dietary inclusion level.

### 2.3. Experiment 1: Intake and Palatability Trial

#### 2.3.1. Animals, Housing, Experimental Design, and Diets

Intake and palatability were evaluated in June 2013 using the cafeteria technique, which allows voluntary selection among feeds containing secondary compounds to be assessed [29,30]. The trial lasted 24 d, including 16 d of adaptation followed by 8 d of data collection. During adaptation, lambs were housed individually and simultaneously exposed to the five experimental diets using the same cafeteria feeder and feeding schedule subsequently applied during data collection. This period allowed the animals to become familiar with the housing, handling, feeder, and experimental diets before dietary preference was evaluated.

Eight 5-month-old Rambouillet lambs with an initial body weight (IBW) of 40.7 ± 0.2 kg and no previous exposure to dietary sources of CT were used. The number of animals was consistent with previous cafeteria trials conducted in sheep [17,18,30]. Lambs were housed individually in 1.5 × 1.5 m pens equipped with shade, a feeder, and an automatic waterer, with free access to water. Previous exposure was considered because browsing experience can modify the ingestive response to phenolic compounds [31].

Treatments consisted of five diets containing a constant forage fraction of 80% of DM, within which CTWB progressively replaced CS (Table 1). Diets CTWB-0, CTWB-20, CTWB-40, CTWB-60, and CTWB-80 contained 0, 20, 40, 60, and 80% CTWB and 80, 60, 40, 20, and 0% CS, respectively. The remaining ingredients were held constant across diets: 12% soybean meal, 5% cane molasses, 1% urea, 1% monocalcium phosphate, 0.9% calcium carbonate, and 0.1% premix. The CTWB and CS were ground as described in Section 2.2, and each complete diet was prepared in a horizontal mixer. Diets were formulated on a DM basis to meet the maintenance requirements of the lambs according to NRC [5]. Consequently, they supplied 0.00, 5.24, 10.48, 15.72, and 20.96 g CT/kg DM, respectively. Each lamb was simultaneously offered all five diets and served as the experimental unit in a cafeteria design involving within-animal comparisons.

Dietary chemical composition was calculated from the proportion of each ingredient and the analyzed or tabular values described in Section 2.2. For CTWB, the analyzed concentrations of DM, CP, NDF, EE, ash, and CT and the ME estimated through in vitro gas production were used. For CS, the analyzed concentrations of DM, CP, NDF, EE, and ash and the tabular energy values reported by NRC [5] were used; the composition of the remaining ingredients was also obtained from this source. Organic matter (OM) was calculated as OM (%) = 100 − ash, whereas the dietary concentrations of calcium (Ca), phosphorus (P), CT, and ME were obtained from the proportional contribution of each ingredient. The calcium-to-phosphorus ratio was calculated as Ca/P, and TDN, NE_m_, and NE_g_ were estimated from ME. Ingredient composition and the calculated chemical and energy values of the diets are presented in Table 1.

Each pen was equipped with a cafeteria feeder divided into five independent compartments, each containing 0.10 kg DM of one experimental diet. Diet positions within the feeder were randomized daily to minimize location-related preferences [32]. Dietary preference was evaluated before the morning feeding, after which lambs received alfalfa hay at 1000 and 2000 h, with a total daily allowance of 20 g DM/kg body weight (BW). This procedure was used to limit hunger-driven intake and promote discrimination among the experimental diets [17,18,30].

Individual pens allowed visual and auditory contact with neighboring lambs; no additional environmental enrichment was provided.

#### 2.3.2. Intake and Palatability Measurements

Dietary preference was evaluated daily from 0830 to 0900 h during the 8-d data collection period. At the end of each evaluation, refusals from each compartment were collected and weighed separately. For each lamb, diet, and day, DMI was calculated on a DM basis as DMI (kg DM) = DM offered − DM refused. The proportion of diet consumed (PDC) was calculated as PDC (%) = (DMI of each diet/DM offered for the same diet) × 100, whereas the relative palatability index (RPI) was estimated according to Abdulrazak et al. [29] as RPI (%) = (DMI of each diet/DMI of the most consumed diet) × 100. Daily PDC and RPI values were averaged for each lamb across the 8-d evaluation period.

### 2.4. Experiment 2: Growth Performance Trial

#### 2.4.1. Animals, Housing, Experimental Design, and Diets

Thirty-six intact purebred Rambouillet ram lambs selected as prospective breeding rams were used. The lambs were 6 months old, had an IBW of 49.97 ± 0.79 kg, and had a body condition score of 2.5 on a 1-to-5 scale. Sample size was established based on the availability of individual pens and previous experiments with similar designs and growth variables. Before the experiment, the animals were individually identified, dewormed, and vaccinated subcutaneously against pneumonic pasteurellosis with 2 mL of One Shot^®^ (Zoetis México, Mexico City, Mexico). Lambs were then adapted to the housing, handling, and experimental diets for 15 d, followed by a 70 d evaluation period.

Lambs were randomly assigned by drawing lots from a lottery drum to one of four dietary treatments, with nine animals per treatment, according to a completely randomized design. Randomization was not stratified by IBW. Pen location was randomly assigned to distribute treatments throughout the experimental area, and animal handling and measurement order were also randomized to minimize potential effects associated with pen location and measurement order. Each lamb served as the experimental unit and was housed individually in a fully roofed 3 × 2 m pen equipped with a metal feeder and a manually filled waterer. Animals had continuous access to water throughout the adaptation and experimental periods.

Treatments were selected based on the intake and palatability trial and consisted of progressively replacing CS with CTWB within a constant forage fraction representing 60% of dietary DM. Diets CTWB-0, CTWB-20, CTWB-40, and CTWB-60 contained 0, 20, 40, and 60% CTWB and 60, 40, 20, and 0% CS, respectively (Table 2). The remaining ingredients were held constant across diets: 20% steam-flaked corn, 12% soybean meal, 5% cane molasses, 1% urea, 1% monocalcium phosphate, 0.9% calcium carbonate, and 0.1% premix. Consequently, the diets supplied 0.00, 5.24, 10.48, and 15.72 g CT/kg DM, respectively. All diets were formulated on a DM basis to meet the nutrient requirements of growing lambs established by NRC [5].

Chemical composition and energy values were calculated using the procedure described in Section 2.3.1 and are presented in Table 2. Diets were offered at 0900 and 1600 h, with the daily allowance divided equally between feedings. The initial feed allowance was set at 3.0% of IBW on a DM basis and subsequently adjusted daily in 5% increments or reductions according to the amount refused on the previous day to maintain *ad libitum* intake.

Individual pens allowed visual and auditory contact with neighboring lambs; no additional environmental enrichment was provided.

#### 2.4.2. Growth Performance and Feed Efficiency

Lambs were weighed individually at the beginning and end of the 70 d experimental period to determine IBW and final body weight (FBW), respectively. Weighing was conducted before the morning feeding after a 12-h withdrawal from solid feed, using an electronic scale with a capacity of 200 kg (CRS-200, Torrey, Monterrey, Nuevo León, Mexico). Cumulative body weight change (CBWC) was calculated as CBWC (kg) = FBW − IBW, and average daily gain (ADG) as ADG (kg/d) = CBWC/70.

The amounts of feed offered and refused were recorded daily for each lamb. Daily DMI was calculated as DMI (kg DM/d) = DM offered − DM refused and subsequently averaged over the 70 d experimental period. Feed efficiency was expressed as the gain-to-feed ratio (G:F) and calculated as G:F = ADG/DMI.

#### 2.4.3. Energy Utilization Calculations

Energy utilization was estimated individually from the BW, ADG, and DMI observed during the 70 d experimental period according to Zinn et al. [33]. Shrunk body weight (SBW) was calculated as SBW (kg) = 0.96 × [(IBW + FBW)/2]. Daily energy requirements for maintenance (EM) and gain (EG) were subsequently estimated as EM (Mcal/d) = 0.077 × SBW^0.75^ and EG (Mcal/d) = 0.0608 × SBW^0.75^ × ADG^1.097^, respectively.

Observed dietary NE_m_ was obtained by iteratively solving the relationship DMI = (EM/NE_m_) + (EG/NE_g_), where NE_g_ = 0.877 × NE_m_ − 0.41. Observed dietary NE_g_ was then calculated from the resulting NE_m_ using the latter equation. Expected DMI was estimated using the calculated dietary NE_m_ and NE_g_ values presented in Table 2 as expected DMI (kg DM/d) = (EM/calculated dietary NE_m_) + (EG/calculated dietary NE_g_).

The observed-to-expected DMI, NE_m_, and NE_g_ ratios were calculated by dividing each observed value by its corresponding expected or calculated value: observed DMI/expected DMI, observed dietary NE_m_/calculated dietary NE_m_, and observed dietary NE_g_/calculated dietary NE_g_, respectively. Finally, the difference between observed DMI and that expected from the calculated energy concentration of each diet was estimated as observed DMI − energy-expected DMI (kg DM/d) = observed DMI − expected DMI.

#### 2.4.4. Ultrasound Measurements

Rib fat thickness (FAT; mm) and longissimus muscle area (LMA; cm^2^) were measured between the 12th and 13th ribs on the left side using real-time ultrasonography at the end of the experimental period, after a 12-h withdrawal from solid feed. Before scanning, the wool was removed, the measurement site was cleaned with compressed air, and acoustic gel was applied. Images were obtained using an Aloka ProSound 2 ultrasound unit equipped with a 3.5-MHz linear transducer (Hitachi Aloka Medical, Tokyo, Japan). The transducer was positioned perpendicular to the vertebral column to obtain a cross-sectional image of the longissimus muscle. Rib fat thickness was measured as the depth of subcutaneous adipose tissue overlying the muscle, whereas LMA was determined by tracing the muscle perimeter. All images were obtained and evaluated by the same trained technician, who was blinded to the dietary treatment assigned to each lamb.

#### 2.4.5. Scrotal Development and Semen Characteristics

Scrotal circumference (SC) was measured on d 0, 14, 28, 42, 56, and 70 according to Fuenmayor et al. [34]. The d 0 evaluation was considered the baseline measurement. For each measurement, the head and tail of the epididymis were identified, both testes were firmly displaced toward the bottom of the scrotum, and a flexible measuring tape was placed around the region of greatest circumference. Total change in SC was calculated as total change in SC (cm) = SC on d 70 − SC on d 0.

On the same sampling days, semen samples were collected from each lamb by electroejaculation. Animals were physically restrained, and feces were removed from the rectum before probe insertion. Semen was collected using a portable ElectroJac^®^ 6 electroejaculator (Ideal Instruments, Neogen Corporation, Lexington, KY, USA) equipped with a rectal probe for rams measuring 25 mm in diameter and 171 mm in length (Ram/Boar Probe, Model 6989). Electrical stimulation was progressively increased until ejaculation. The ejaculate was collected through a plastic cone attached to a sterile, graduated 15-mL conical tube. Samples were maintained at approximately 37 °C during transport to the Animal Reproduction Laboratory of UAMVZ-UAZ and evaluated within 30 min of collection [35,36]. Sperm concentration was determined immediately after collection using a Neubauer hemocytometer (BRAND GmbH + Co. KG, Wertheim, Germany) and adjusted to 1600 × 10^6^ spermatozoa/mL with Tris-based extender before splitting the sample for viability, morphology, and motility assessments.

Sperm viability and morphology were evaluated using eosin–nigrosin staining by mixing semen and staining solution at a 1:1 ratio. A smear was prepared from each sample and examined using an upright light microscope (DM4 B, Leica Microsystems GmbH, Wetzlar, Germany) with a 40× objective. For viability assessment, 50 sperm cells per sample were evaluated across different fields of the smear. Cells that excluded eosin were classified as viable, whereas those stained pink or red were considered nonviable. In an independent count of 50 cells, sperm were classified as normal or abnormal based on visible alterations of the head, midpiece, or tail, following a procedure adapted from Palacin-Martinez et al. [37].

Sperm motility parameters were evaluated using a computer-assisted sperm analysis system (CASA; IVOS, Version 12, Hamilton-Thorne Biosciences, Beverly, MA, USA) configured for ram semen. Immediately before analysis, 50 μL of the concentration-adjusted sample was mixed with 950 μL of Tris-based extender to obtain a final dilution of 1:20. Subsequently, 10 μL of the diluted sample was loaded into a four-chamber Leja slide with a depth of 20 μm (Leja Products B.V., Nieuw-Vennep, The Netherlands), prewarmed to 37 °C. At least 400 sperm cells were analyzed per sample. Total, progressive, and non-progressive motility and the proportion of immotile sperm were expressed as percentages of all cells analyzed. All semen evaluations were performed by the same trained technician, who remained blinded to the dietary treatment assigned to each animal.

#### 2.4.6. Economic Evaluation

The economic evaluation was conducted individually for all 36 lambs over the 70-d experimental period using DMI, CBWC, and the cost of the diet assigned to each animal. Diet cost was calculated on a DM basis from the inclusion rate and unit price of each ingredient and was 0.225, 0.210, 0.194, and 0.179 USD/kg DM for CTWB-0, CTWB-20, CTWB-40, and CTWB-60, respectively. All monetary values were expressed in USD. Ingredient prices and the live BW selling price reflected current market conditions at the time of manuscript preparation rather than prices from the original experimental period.

Total feed cost was calculated as total feed cost (USD/lamb) = DMI (kg DM/d) × 70 d × diet cost (USD/kg DM). Income from BW gain was estimated using a live BW selling price of 4.78 USD/kg and calculated as income from BW gain (USD/lamb) = CBWC (kg) × 4.78 USD/kg BW. Income over feed cost (IOFC) was then calculated as IOFC (USD/lamb) = income from BW gain − total feed cost. Finally, cost of gain (COG) was calculated as COG (USD/kg BW gain) = total feed cost/CBWC. All economic indicators were calculated for each lamb and subsequently averaged by treatment.

### 2.5. Statistical Analysis

The experiments were analyzed independently using SAS^®^ OnDemand for Academics: SAS Studio, version 3.82 (SAS Institute Inc., Cary, NC, USA) [38]. Residual normality and homogeneity of variance were assessed using the Shapiro–Wilk and Levene tests, respectively. For the intake and palatability trial, daily DMI, PDC, and RPI were averaged for each lamb across the 8 d and analyzed using the mixed model *Y_ij_* = *μ* + *D_i_* + *A_j_* + *ε_ij_*, where *D_i_* was the fixed effect of diet and *A_j_* the random effect of lamb. The growth trial was analyzed as a completely randomized design, with dietary treatment as the fixed effect and lamb as the experimental unit. Variables requiring covariate adjustment were analyzed as $Y_{\mathrm{ij}}=\mu +T_{i}+\beta \left(X_{\mathrm{ij}}-\bar{X}\right)+\varepsilon_{\mathrm{ij}}$. No a priori power analysis was performed. The sample size for Experiment 1 was based on previous cafeteria trials in sheep, whereas that for Experiment 2 was determined from the availability of individual pens and previous experiments with similar designs and growth variables. Because no primary outcome was prespecified for sample-size determination, the evaluated responses were considered exploratory. Initial BW was used as a covariate for FBW and initial SC for final SC and SC change. Scrotal circumference and semen characteristics measured on d 14, 28, 42, 56, and 70 were analyzed separately using ANCOVA, with dietary treatment as the fixed effect and the corresponding d 0 measurement as a covariate. All remaining variables were analyzed without a covariate, and initial BW and SC were analyzed by ANOVA.

The PDC and RPI were analyzed on the original percentage scale because model assumptions were satisfied. Sperm viability, morphologically abnormal sperm, and CASA-derived motility variables were also evaluated after arcsine square-root transformation; because the conclusions were unchanged, least squares means and standard error of the mean (SEM) are reported on the original scale. For the economic evaluation, IOFC and COG were calculated for each lamb and analyzed by one-way ANOVA with dietary treatment as the fixed effect. Treatment means for all response variables, including IOFC and COG, were compared using Tukey’s test within each response variable. No additional adjustment for multiplicity across response variables was applied. Results are presented as least squares means and SEM, and differences were considered significant at *p* ≤ 0.05.

Personnel responsible for diet preparation and feeding were aware of treatment allocation because the experimental diets were visually distinguishable. In contrast, the technicians responsible for ultrasound and semen evaluations were blinded to dietary treatment, and statistical analyses were conducted using treatment codes.

## 3. Results

### 3.1. Intake and Palatability Measurements

Condensed tannin-containing woody biomass replaced 20 percentage units of the diet originally supplied by CS, reducing CS from 80 to 60%, without affecting PDC or RPI compared with CTWB-0 (*p* > 0.05; Table 3). Diet CTWB-20 supplied 5.24 g CT/kg DM and resulted in a PDC of 75.31% and an RPI of 100.00%, compared with 68.47 and 95.21% for CTWB-0, respectively. In contrast, CTWB inclusions of 40 and 60%, which supplied 10.48 and 15.72 g CT/kg DM, reduced PDC to 49.34 and 46.25% and RPI to 66.87 and 64.75%, respectively, compared with CTWB-0 and CTWB-20 (*p* ≤ 0.05). Complete replacement of CS with CTWB-80, equivalent to 20.96 g CT/kg DM, resulted in the lowest PDC (31.33%; *p* ≤ 0.05) and an RPI of 43.72%, which was lower than those of CTWB-0, CTWB-20, and CTWB-40 but similar to that of CTWB-60. Overall, increasing CT concentrations supplied through CTWB affected PDC and RPI (*p* = 0.014 and *p* = 0.015, respectively); however, 20% CTWB was the highest inclusion level that replaced CS without reducing proportional intake or relative palatability.

### 3.2. Growth Performance and Feed Efficiency

In the growth trial, the inclusion of 20% CTWB, which supplied 5.24 g CT/kg DM, reduced CS from 60 to 40% and produced a better growth response than complete CS replacement with CTWB-60 (Table 4). Lambs fed CTWB-20 had greater FBW (63.95 vs. 57.87 kg; *p* = 0.016), ADG and CBWC (0.202 vs. 0.126 kg/d and 14.19 vs. 8.93 kg, respectively; *p* = 0.019 for both variables), and G:F (0.094 vs. 0.058; *p* = 0.011) than those fed CTWB-60, which supplied 15.72 g CT/kg DM. Treatments CTWB-0 and CTWB-40, the latter supplying 10.48 g CT/kg DM, had intermediate values and did not differ from either CTWB-20 or CTWB-60 (*p* > 0.05). Despite these differences in growth, DMI remained similar among treatments (2.00–2.18 kg DM/d; *p* = 0.278); therefore, the greater gain and G:F observed with CTWB-20 were not accompanied by increased feed intake. Overall, including 20% CTWB replaced one-third of the CS originally present in the diet, maintained DMI, and supported greater growth and G:F than complete CS replacement.

### 3.3. Energy Utilization Calculations and Ultrasound Measurements

Progressive replacement of CS with CTWB did not affect observed dietary NE_m_ or NE_g_ concentrations, even when CTWB-60 completely replaced CS and supplied 15.72 g CT/kg DM (*p* = 0.146 for both variables; Table 5). However, the decline in calculated dietary energy concentration as CTWB increased raised expected DMI from 1.081 kg DM/d with CTWB-0 to 1.502 kg DM/d with CTWB-60, whereas CTWB-20 and CTWB-40 had intermediate values (*p* < 0.0001). Consequently, the observed-to-expected DMI ratio was lower with CTWB-40 and CTWB-60 than with CTWB-0, whereas CTWB-20 had an intermediate response (*p* = 0.003). In contrast, the observed-to-expected NE_m_ ratio was greater with CTWB-40 and CTWB-60 than with CTWB-0, and CTWB-60 also exceeded CTWB-20 (*p* = 0.002); for the observed-to-expected NE_g_ ratio, only CTWB-60 exceeded CTWB-0 (*p* = 0.045). These responses were associated with the lower calculated energy values used as denominators rather than with an increase in observed NE_m_ or NE_g_ concentrations. Additionally, the difference between observed and expected DMI remained similar among treatments (*p* = 0.141).

Including 20% CTWB, equivalent to 5.24 g CT/kg DM, reduced CS from 60 to 40% without decreasing FAT relative to CTWB-0 (2.06 vs. 2.22 mm; *p* > 0.05). In contrast, diets CTWB-40 and CTWB-60, which supplied 10.48 and 15.72 g CT/kg DM, resulted in lower FAT than CTWB-0 (1.92 and 1.81 vs. 2.22 mm, respectively; *p* = 0.004), although neither differed from CTWB-20. Longissimus muscle area remained similar among treatments (10.35–11.42 cm^2^; *p* = 0.157). Overall, including 20% CTWB maintained observed dietary energy, FAT, and LMA, whereas inclusion levels of 40% or greater maintained observed NE_m_ and NE_g_ concentrations and LMA but reduced FAT.

### 3.4. Scrotal Development and Semen Characteristics

Partial or complete replacement of CS with CTWB did not affect scrotal development during the 70-d evaluation period. Final SC and its change during the experiment were similar among treatments (*p* = 0.167 for both variables; Table 6). Across the intermediate evaluations, dietary treatment affected morphologically abnormal sperm on d 14 (*p* = 0.028), non-progressive motility on d 28 (*p* = 0.014), and sperm viability on d 56 (*p* = 0.031). No treatment effects were detected for the remaining reproductive variables during these evaluations (*p* > 0.05; Table S1). At the final evaluation, the inclusion of 20% CTWB, equivalent to 5.24 g CT/kg DM, increased sperm viability from 49.30% in CTWB-0 to 87.48% in CTWB-20 (*p* = 0.032). CTWB-40 and CTWB-60, which supplied 10.48 and 15.72 g CT/kg DM, respectively, showed intermediate values of 81.06 and 78.26% and did not differ from either CTWB-0 or CTWB-20 (*p* > 0.05). Additionally, all CTWB inclusion levels reduced the proportion of morphologically abnormal sperm from 45.78% in CTWB-0 to 13.32, 23.56, and 5.78% in CTWB-20, CTWB-40, and CTWB-60, respectively (*p* = 0.005). Total and progressive motility, non-progressive motility, and the proportion of immotile sperm remained similar among treatments at the final evaluation (*p* = 0.161, *p* = 0.372, *p* = 0.408, and *p* = 0.240, respectively). Overall, the inclusion of 20% CTWB reduced CS from 60 to 40% and was associated with greater sperm viability and fewer morphological abnormalities than CTWB-0, without affecting scrotal development or final sperm motility. Although the higher CTWB inclusion levels did not significantly increase viability relative to CTWB-0, they reduced morphological abnormalities and maintained the remaining reproductive indicators at the final evaluation.

### 3.5. Economic Evaluation

The growth response obtained with CTWB-20 numerically translated into more favorable economic returns (Table 7). Although treatment did not affect IOFC (*p* = 0.217) or COG (*p* = 0.735), CTWB-20 showed the highest IOFC (36.20 USD/lamb) and the lowest COG (2.55 USD/kg BW gain), whereas CTWB-60 showed the lowest IOFC (15.33 USD/lamb). Increasing CTWB reduced diet cost and total feed cost; however, these reductions did not translate into improved economic efficiency at the higher inclusion levels.

## 4. Discussion

### 4.1. Intake and Palatability Measurements

The principal finding of the preference trial was that including 20% CTWB, equivalent to 5.24 g CT/kg DM, partially replaced CS without reducing PDC or RPI. This response agrees with Carrillo-Muro et al. [17], who included 10–30% *Q. rugosa* CTWB, equivalent to 2.4–7.3 g CT/kg DM, without affecting either variable. However, an inclusion of 40% and 9.7 g CT/kg DM produced an intermediate response, whereas 50–70% and 12.2–17.0 g CT/kg DM reduced acceptance. Together, both studies indicate that acceptance of *Q. rugosa* CTWB is maintained at moderate inclusion levels but begins to decline at approximately 40% of the diet and 10 g CT/kg DM. Nevertheless, this response is not consistent across *Quercus* species. Carrillo-Muro et al. [18] reported that up to 40% *Q. resinosa* CTWB, equivalent to approximately 16 g CT/kg DM, maintained an RPI similar to that of the CTWB-free diet, although the proportion consumed decreased at 20% inclusion and the remaining levels produced intermediate responses. Lambs also preferred material collected in spring, indicating that plant species, collection season, and nutritional composition contribute to dietary acceptance.

The lower PDC and RPI observed at the highest CTWB levels also agree with responses to other tannin-containing sources included at high proportions. Priolo et al. [39] reported that a diet containing 56% carob pulp and 25 g CT/kg DM reduced DMI, growth, digestibility, and G:F relative to the same diet supplemented with polyethylene glycol (PEG). Similarly, Vasta et al. [40] observed lower DMI and ADG with 45% carob pulp and 27 g CT/kg DM, effects that were counteracted by PEG. These responses are consistent with the lower acceptance of CTWB-80, which supplied 20.96 g CT/kg DM; however, similar concentrations do not always produce the same effect. Bonanno et al. [41] observed greater DMI and growth with sulla supplying 20.3 g CT/kg DM than with CT-free ryegrass, a response also associated with the greater CP and nonstructural carbohydrate content of sulla. Therefore, dietary acceptance depends not only on CT concentration but also on tannin structure and reactivity, the nutritional value of the source, and its proportion in the diet [9,12].

In the present study, CT increased simultaneously with the replacement of CS by CTWB, thereby modifying both the nutritional composition and sensory characteristics of the diets. Consequently, the reduction in acceptance cannot be attributed exclusively to CT. Nevertheless, agreement with inclusion levels previously evaluated for *Q. rugosa* supports a combined effect of the biomass and its CT content. From an applied perspective, 20% CTWB and 5.24 g CT/kg DM represented the highest level that reduced CS from 80 to 60% without compromising proportional intake or relative palatability.

### 4.2. Growth Performance and Feed Efficiency

Including 20% CTWB, equivalent to 5.24 g CT/kg DM, maintained DMI and resulted in greater FBW, ADG, CBWC, and G:F than complete CS replacement with CTWB-60. Because ADG and G:F are both mathematically derived from CBWC, these four variables should be interpreted as reflecting a single underlying growth response rather than four independent outcomes. However, these values were similar to those obtained with CTWB-0 and CTWB-40; therefore, the results do not demonstrate that CTWB-20 stimulated growth relative to the diet without CTWB. Instead, they indicate that a moderate inclusion replaced one-third of the CS without compromising growth performance. In contrast, complete replacement with 60% CTWB and 15.72 g CT/kg DM did not maintain the productive response achieved with CTWB-20. Because DMI remained similar, this difference reflected lower gain per unit of feed consumed rather than restricted intake. However, progressive replacement of CS with CTWB also reduced dietary CP and calculated energy density; therefore, the lower growth response observed with CTWB-60 cannot be attributed specifically to CT.

Growth responses to tannin-containing sources depend on their composition, inclusion level, and tannin type. Bonanno et al. [41] observed greater DMI, growth, and G:F in lambs consuming sulla with 20.3 g CT/kg DM than in those fed ryegrass; however, this response was also associated with greater CP and nonstructural carbohydrate intake. Valenti et al. [42] included 4% mimosa extract, equivalent to approximately 22.4 g total tannins/kg DM consumed, without affecting DMI, ADG, or FBW, whereas the same inclusion level of chestnut extract reduced these variables. In contrast, diets containing either 56% carob pulp and 25 g CT/kg DM [39] or 45% of this byproduct and 27 g CT/kg DM [40] reduced DMI, ADG, and G:F, and PEG counteracted some of these effects. Hervás et al. [43] also demonstrated that increasing levels of quebracho extract can reduce CP digestibility and, at the highest inclusion, the ruminal disappearance of DM, N, and NDF. Although digestibility was not measured in the present study, these findings indicate that greater exposure to tannins can modify nutrient availability.

The greater G:F observed for CTWB-20 relative to CTWB-60 indicates a more favorable productive use of feed at the moderate inclusion level. Overall, the results support a moderate inclusion of 20% CTWB and 5.24 g CT/kg DM, whereas increasing the biomass to 60% did not maintain the productive advantages achieved at this level.

### 4.3. Energy Utilization Calculations and Ultrasound Measurements

Progressive replacement of CS with CTWB did not affect observed NE_m_ or NE_g_ concentrations, indicating that diets supplying up to 15.72 g CT/kg DM yielded similar dietary energy estimates based on growth and intake. However, the decline in calculated dietary energy concentration as CTWB increased raised expected DMI and consequently reduced the observed-to-expected DMI ratio with CTWB-40 and CTWB-60. In contrast, the observed-to-expected NE_m_ and NE_g_ ratios reached their greatest values with CTWB-60 because the lower calculated energy concentrations reduced the reference values used in these comparisons. Therefore, these ratios indicate closer proportional agreement between observed and calculated energy values rather than improved energy utilization, as the observed concentrations did not increase and CTWB-60 did not maintain the productive response achieved with CTWB-20.

Energy responses to tannins depend on their source, concentration, and duration of supplementation. Rojas-Román et al. [44] supplied 2–6 g extract/kg DM for 70 d, equivalent to 1.4–4.2 g total tannins/kg DM, and observed a transient improvement in estimated dietary energy with the intermediate dose. However, over the complete feeding period, the highest inclusion reduced this variable without affecting ADG, DMI, or G:F. Similarly, Rivera-Méndez et al. [45] supplied approximately 2.2 and 4.4 g CT/kg DM through an extract included at 0.32 and 0.64% of the diet. Both concentrations increased DMI but reduced G:F and estimated NE_m_ and NE_g_ concentrations without affecting ADG; thus, greater intake did not result in greater growth. These studies demonstrate that moderate tannin concentrations can alter dietary energy estimates without producing corresponding changes in gain. Nevertheless, both studies used extracts included at low proportions and maintained a nearly constant basal diet, whereas CTWB represented 20–60% of DM and progressively replaced CS in the present experiment. Consequently, the responses reflect both increasing CT concentrations and concurrent changes in dietary composition and energy concentration and cannot be attributed exclusively to tannins.

Final ultrasound measurements showed that CTWB-20 maintained FAT relative to CTWB-0, whereas CTWB-40 and CTWB-60 reduced FAT without affecting LMA. Keddam et al. [46] observed a partially similar response when 50% barley was replaced with 50% *Q. ilex* acorns, which reduced carcass fat thickness without affecting BW or gain. However, abdominal fat remained similar, whereas intramuscular lipid content increased. In contrast, Rojas-Román et al. [44] and Rivera-Méndez et al. [45] detected no changes in fat thickness or longissimus muscle area with extracts supplying up to 4.2 g total tannins or 4.4 g CT/kg DM, respectively. These differences may be related to the proportion of each source within the diet because the extracts were included at low levels, whereas acorns and CTWB replaced substantial proportions of other ingredients. Furthermore, Keddam et al. [46] did not determine CT concentrations, and their diets differed in fat, fiber, starch, and calculated energy. Therefore, the lower FAT observed with CTWB-40 and CTWB-60 should be interpreted as a response to the complete diet rather than an isolated effect of tannins.

This interpretation is also consistent with the contrasting responses reported for other tannin-containing feed resources. Priolo et al. [39] found that a diet containing 56% carob pulp and 25 g CT/kg DM reduced carcass fat together with digestibility, growth, and G:F. In contrast, Bonanno et al. [41] observed greater growth and fat deposition with sulla containing 20.3 g CT/kg DM, although this response was accompanied by greater DM, CP, and nonstructural carbohydrate intake. Therefore, body fat reflects the interaction among CT source, nutritional value, intake, and growth. In the present study, moderate inclusion of CTWB-20 maintained observed dietary energy concentrations, FAT, and LMA, whereas higher inclusion levels maintained observed energy and muscle area but not subcutaneous fat.

### 4.4. Scrotal Development and Semen Characteristics

Partial or complete replacement of CS with CTWB did not affect final SC or its change during the experiment. Initial SC and IBW did not differ among treatments (*p* = 0.996 and *p* = 0.269, respectively; Table 4 and Table 6), confirming that randomization achieved adequate baseline balance for these covariates. This response was consistent with the similar DMI and observed NE_m_ and NE_g_ concentrations among treatments, suggesting that dietary differences were insufficient to alter scrotal growth. Kheradmand et al. [47] observed greater SC, sperm concentration, and total sperm production when rams fed at maintenance received greater amounts of DM, energy, and CP. Similarly, Fourie et al. [4] reported greater scrotal and testicular development under an intensive system than under extensive conditions, although this response was accompanied by greater scrotal fat deposition and lower sperm motility. Bester et al. [48] also found that increasing dietary energy concentration from 1.97 to 2.71 Mcal ME/kg DM enhanced testicular development and scrotal fat deposition but did not affect semen quantity or quality. These findings indicate that scrotal development may respond to substantial differences in nutritional plane, although greater size does not necessarily imply better semen quality. Therefore, the maintenance of SC indicates that CTWB did not compromise scrotal development but does not demonstrate a stimulatory effect of CT.

In contrast, CTWB-20 was associated with greater sperm viability, and all CTWB levels were associated with fewer morphological abnormalities, without these responses being associated with greater scrotal growth or observed energy supply. Sperm cells are susceptible to oxidative damage because of the high polyunsaturated fatty acid content of their membranes; thus, phenolic compounds in CTWB may have contributed to preserving cellular integrity. Zhao et al. [10] observed that including 5 and 10% grape pomace increased sperm motility and testicular antioxidant enzyme activity, whereas 10% also reduced sperm deformities and improved acrosomal integrity. However, that study did not report the dietary CT concentration, and the present experiment did not measure antioxidant capacity, reactive oxygen species, or lipid peroxidation. Consequently, an antioxidant effect is biologically plausible but cannot be confirmed or attributed exclusively to CT. The relatively low viability observed in CTWB-0 should be interpreted cautiously, because each sampling time was represented by a single electroejaculate per ram, and semen characteristics may have been influenced by individual variation associated with sexual maturity and the collection procedure. To our knowledge, no directly comparable reference values for sperm viability in Rambouillet ram lambs of similar age have been reported, and this baseline value should therefore be interpreted relative to the other treatment groups evaluated under identical conditions rather than against external reference ranges.

Sperm viability was greatest with CTWB-20, whereas CTWB-40 and CTWB-60 had intermediate values, indicating that increasing CT above 5.24 g/kg DM did not further modify this response. In contrast, the reduction in abnormalities at all CTWB levels indicates that sperm morphology responded across a broader inclusion range. Total, progressive and non-progressive motility, and the proportion of immotile sperm did not differ significantly among treatments; however, given the high residual variability associated with a single ejaculate per ram at each sampling time, these traits had limited statistical power to detect treatment effects of the magnitude observed for viability and morphology, and the absence of significance should not be interpreted as confirming that sperm movement was unaffected. These variables represent different components of semen quality and may respond independently, as also reported by Kheradmand et al. [47] and Bester et al. [48]. Overall, including 20% CTWB and 5.24 g CT/kg DM reduced CS from 60 to 40% without compromising scrotal development or sperm motility and was associated with greater sperm viability and fewer morphological abnormalities than CTWB-0.

### 4.5. Economic Evaluation

Including 20% CTWB allowed partial replacement of CS without adversely affecting economic efficiency. Although DMI and total feed cost were similar to those of CTWB-0, IOFC and COG were also maintained, indicating that the lower-cost fiber source could be incorporated without an economic penalty at this inclusion level. In contrast, CTWB-40 and CTWB-60 reduced diet cost and total feed cost, but these savings did not offset the lower income from BW gain. Thus, reducing diet cost through greater inclusion of a fiber source does not ensure an economic advantage unless growth performance is maintained.

These results emphasize the importance of identifying an appropriate replacement level rather than maximizing CTWB inclusion. Including 20% CTWB, equivalent to 5.24 g CT/kg DM, reduced CS from 60 to 40% and allowed the use of a lower-cost fiber source while maintaining productive performance and economic efficiency comparable with the control diet. Conversely, higher inclusion levels reduced feeding costs but did not maintain the productive response observed at the lower replacement level. Under the prices and conditions evaluated, partial replacement of CS with 20% CTWB appears feasible without negatively affecting overall economic efficiency.

### 4.6. Study Limitations

This study has several limitations. Progressive replacement of CS with CTWB simultaneously increased CT concentration and altered the nutritional composition of the diets, including progressive reductions in CP, NDF, and calculated energy density; therefore, the specific effects of tannins could not be separated from those associated with replacement of the fiber source and the accompanying changes in nutrient composition. Condensed tannins were not quantified in CS; consequently, the reported dietary CT gradient represents the calculated CT contribution from CTWB rather than analytically determined total dietary CT concentrations. The energy contribution of CTWB and CS was derived using different approaches, with CTWB ME estimated from in vitro gas production and CS energy values obtained from NRC tabular data. Therefore, the calculated dietary energy values and the corresponding O:E ratios should be interpreted as reference estimates rather than as measurements derived from a uniform analytical method. The CTWB used in Experiment 2 originated from the same batch collected in 2013 and was not reanalyzed before the growth trial. Although it was stored under dry conditions protected from light, moisture, and pests, possible changes in CT concentration or reactivity during storage cannot be excluded. In addition, the economic evaluation depended on the prices used and did not include a sensitivity analysis of changes in ingredient costs or the live BW price. Semen was collected on d 0, 14, 28, 42, 56, and 70; treatment differences were detected for selected semen characteristics on d 14, 28, 56, and 70, whereas most outcomes were similar among treatments (Table S1). However, each sampling time was represented by a single ejaculate per ram, and the relatively young age of the animals and the moderate sample size may have limited the precision and statistical power for some semen characteristics, particularly sperm motility variables. Finally, the results apply to prospective Rambouillet breeding ram lambs fed *Q. rugosa* CTWB; therefore, extrapolation to other breeds, production stages, *Quercus* species, tannin sources, or feeding systems should be approached with caution.

## 5. Conclusions

Under the conditions of this study, including 20% CTWB, corresponding to a calculated CT contribution from CTWB of 5.24 g/kg DM, partially replaced CS without reducing dietary acceptance or intake. In the growth trial, this inclusion reduced CS from 60 to 40% and maintained growth performance comparable with CTWB-0 while supporting greater growth and G:F than complete CS replacement with CTWB-60, without compromising observed dietary energy, FAT, LMA, or scrotal development. It was also associated with greater sperm viability and fewer sperm abnormalities while maintaining economic efficiency without an adverse effect on IOFC or COG. Overall, the results support including 20% *Q. rugosa* CTWB as an alternative to partially replace CS in diets for prospective Rambouillet breeding ram lambs. In contrast, higher inclusion levels reduced palatability and did not maintain the productive response observed with CTWB-20; therefore, complete CS replacement was not recommended under the conditions evaluated.

## Figures and Tables

**Table 1:** Ingredient and calculated chemical composition of diets containing increasing levels of condensed tannin-containing woody biomass (CTWB) used in the cafeteria preference trial.

Ingredients, % DM	Condensed Tannins from CTWB, g/kg DM ^1^				
	0.00	5.24	10.48	15.72	20.96
	Treatments ^2^				
	CTWB-0	CTWB-20	CTWB-40	CTWB-60	CTWB-80
Corn stover	80.00	60.00	40.00	20.00	0.00
Condensed tannin-containing woody biomass	0.00	20.00	40.00	60.00	80.00
Soybean meal (44% crude protein)	12.00	12.00	12.00	12.00	12.00
Cane molasses	5.00	5.00	5.00	5.00	5.00
Urea	1.00	1.00	1.00	1.00	1.00
Monocalcium phosphate	1.00	1.00	1.00	1.00	1.00
Calcium carbonate	0.90	0.90	0.90	0.90	0.90
Premix ^3^	0.10	0.10	0.10	0.10	0.10
**Chemical composition, % ^4^**					
Dry matter	88.55	87.76	86.96	86.16	85.36
Organic matter	90.81	91.05	91.28	91.51	91.75
Crude protein	14.29	13.76	13.24	12.71	12.18
Neutral detergent fiber	53.79	52.43	51.07	49.71	48.35
Ether extract	1.87	1.76	1.64	1.52	1.41
Ash	9.19	8.96	8.72	8.49	8.25
Calcium	0.95	0.82	0.70	0.58	0.45
Phosphorus	0.39	0.37	0.35	0.33	0.32
Calcium/Phosphorus ratio	2.45	2.23	1.99	1.73	1.43
Condensed tannins, g/kg DM	0.00	5.24	10.48	15.72	20.96
**Calculated energy values ^5^**					
Metabolizable energy, Mcal/kg DM	2.40	2.23	2.06	1.89	1.71
Total digestible nutrients, % DM	66.36	61.62	56.88	52.13	47.39
Net energy for maintenance, Mcal/kg DM	1.53	1.37	1.21	1.04	0.88
Net energy for gain, Mcal/kg DM	0.95	0.80	0.65	0.50	0.34

^1^ Calculated from the analyzed condensed tannin (CT) concentration of CTWB (26.2 g/kg DM) and its dietary inclusion rate; CT in corn stover (CS) was not determined. ^2^ CTWB-0, CTWB-20, CTWB-40, CTWB-60, and CTWB-80 correspond to diets containing 0, 20, 40, 60, and 80% CTWB, respectively, representing progressive replacement of CS by CTWB within a forage fraction comprising 80% of dietary DM. ^3^ Each kg of premix supplied 221.6 mg Co, 125,420 mg Fe, 402.5 mg I, 10,064 mg Mn, 17,078.5 mg Zn, 267 mg Se, 7813 mg Cu, 2,595,000 IU of vitamin A, 468,750 IU of vitamin D, and 28,000 IU of vitamin E. ^4^ Dietary composition was calculated from the proportional contribution of the ingredients. Dry matter is expressed on an as-fed basis; all other components are expressed on a DM basis. Organic matter was calculated as 100 − ash, whereas calcium and phosphorus were calculated using tabular ingredient values from [5]. ^5^ Metabolizable energy was calculated using the estimated ME of CTWB and tabular values for the remaining ingredients; TDN, NE_m_, and NE_g_ were subsequently estimated from ME.

**Table 2:** Ingredient and calculated chemical composition of diets containing increasing levels of condensed tannin-containing woody biomass used in the growth performance trial.

Ingredients, % DM	Condensed Tannins from CTWB, g/kg DM ^1^			
	0.00	5.24	10.48	15.72
	Treatments ^2^			
	CTWB-0	CTWB-20	CTWB-40	CTWB-60
Corn stover	60.00	40.00	20.00	0.00
Condensed tannin-containing woody biomass	0.00	20.00	40.00	60.00
Steam-flaked corn	20.00	20.00	20.00	20.00
Soybean meal (44% crude protein)	12.00	12.00	12.00	12.00
Cane molasses	5.00	5.00	5.00	5.00
Urea	1.00	1.00	1.00	1.00
Monocalcium phosphate	1.00	1.00	1.00	1.00
Calcium carbonate	0.90	0.90	0.90	0.90
Premix ^3^	0.10	0.10	0.10	0.10
**Chemical composition, % ^4^**				
Dry matter	87.95	87.16	86.36	85.56
Organic matter	91.93	92.17	92.40	92.63
Crude protein	14.95	14.42	13.90	13.37
Neutral detergent fiber	42.59	41.23	39.87	38.51
Ether extract	2.31	2.20	2.08	1.96
Ash	8.07	7.84	7.60	7.37
Calcium	0.83	0.71	0.58	0.46
Phosphorus	0.43	0.41	0.40	0.38
Calcium/Phosphorus ratio	1.92	1.71	1.47	1.21
Condensed tannins, g/kg DM	0.00	5.24	10.48	15.72
**Calculated energy values ^5^**				
Metabolizable energy, Mcal/kg DM	2.60	2.42	2.25	2.08
Total digestible nutrients, % DM	71.79	67.05	62.31	57.56
Net energy for maintenance, Mcal/kg DM	1.70	1.53	1.37	1.21
Net energy for gain, Mcal/kg DM	1.09	0.94	0.79	0.64

^1^ Calculated from the analyzed condensed tannin (CT) concentration of CTWB (26.2 g/kg DM) and its dietary inclusion rate; CT in corn stover (CS) was not determined. ^2^ CTWB-0, CTWB-20, CTWB-40, and CTWB-60 correspond to diets containing 0, 20, 40, and 60% condensed tannin-containing woody biomass (CTWB), respectively, representing progressive replacement of CS by CTWB within a forage fraction comprising 60% of dietary DM. ^3^ Each kg of premix supplied 221.6 mg Co, 125,420 mg Fe, 402.5 mg I, 10,064 mg Mn, 17,078.5 mg Zn, 267 mg Se, 7813 mg Cu, 2,595,000 IU of vitamin A, 468,750 IU of vitamin D, and 28,000 IU of vitamin E. ^4^ Dietary composition was calculated from the proportional contribution of the ingredients. Dry matter is expressed on an as-fed basis; all other components are expressed on a DM basis. Organic matter was calculated as 100 − ash, whereas calcium and phosphorus were calculated using tabular ingredient values from [5]. ^5^ Metabolizable energy was calculated using the estimated ME of CTWB and tabular values for the remaining ingredients; TDN, NE_m_, and NE_g_ were subsequently estimated from ME.

**Table 3:** Proportion of diet consumed and relative palatability index of Rambouillet ram lambs offered diets containing increasing levels of condensed tannin-containing woody biomass (CTWB; *n* = 8).

Item	Condensed Tannins from CTWB, g/kg DM ^1^					SEM ^3^	*p*-Value
	0.00	5.24	10.48	15.72	20.96		
	Treatments ^2^						
	CTWB-0	CTWB-20	CTWB-40	CTWB-60	CTWB-80		
Proportion of diet consumed, % ^4^	68.47 ^a^	75.31 ^a^	49.34 ^b^	46.25 ^b^	31.33 ^c^	2.24	0.014
Relative palatability index, % ^5^	95.21 ^a^	100.00 ^a^	66.87 ^b^	64.75 ^bc^	43.72 ^c^	5.48	0.015

^1^ Calculated from the analyzed condensed tannin (CT) concentration of CTWB and its dietary inclusion rate; CT in corn stover (CS) was not determined. ^2^ CTWB-0, CTWB-20, CTWB-40, CTWB-60, and CTWB-80 correspond to diets containing 0, 20, 40, 60, and 80% CTWB, respectively, representing progressive replacement of CS by CTWB. ^3^ SEM = standard error of the mean. Values are presented as least squares means. ^4^ Proportion of diet consumed (PDC; %) = [DMI of each diet (kg DM)/DM offered for that diet (kg DM)] × 100. ^5^ Relative palatability index (RPI; %) = (DMI of each diet/DMI of the most consumed diet) × 100. ^a–c^ Means within a row with different superscripts differ (*p* ≤ 0.05) according to Tukey’s multiple comparison test.

**Table 4:** Growth performance and feed efficiency of Rambouillet ram lambs selected as breeding prospects and fed diets containing increasing levels of condensed tannin-containing woody biomass (CTWB; *n* = 36; 9 lambs/treatment).

Item	Condensed Tannins from CTWB, g/kg DM ^1^				SEM ^3^	*p*-Value
	0.00	5.24	10.48	15.72		
	Treatments ^2^					
	CTWB-0	CTWB-20	CTWB-40	CTWB-60		
Body weight, kg						
Initial	51.23	49.76	49.88	48.99	0.79	0.269
Final	62.80 ^ab^	63.95 ^a^	59.99 ^ab^	57.87 ^b^	1.35	0.016
Average daily gain, kg/d	0.165 ^ab^	0.202 ^a^	0.145 ^ab^	0.126 ^b^	0.02	0.019
Cumulative body weight change, kg	11.57 ^ab^	14.19 ^a^	10.13 ^ab^	8.93 ^b^	1.02	0.019
Dry matter intake, kg DM/d	2.00	2.15	2.18	2.17	0.07	0.278
Gain-to-feed ratio, kg/kg	0.083 ^ab^	0.094 ^a^	0.066 ^ab^	0.058 ^b^	0.01	0.011

^1^ Calculated from the analyzed condensed tannin (CT) concentration of CTWB and its dietary inclusion rate; CT in corn stover (CS) was not determined. ^2^ CTWB-0, CTWB-20, CTWB-40, and CTWB-60 correspond to diets containing 0, 20, 40, and 60% CTWB, respectively, representing progressive replacement of CS by CTWB. ^3^ Standard error of the mean. Values are presented as least squares means. ^a,b^ Means within a row with different superscripts differ (*p* ≤ 0.05) according to Tukey’s multiple comparison test.

**Table 5:** Dietary energy utilization and ultrasound measurements of Rambouillet ram lambs selected as breeding prospects and fed diets containing increasing levels of condensed tannin-containing woody biomass (CTWB; *n* = 36; 9 lambs/treatment).

Item ^1^	Condensed Tannins from CTWB, g/kg DM ^2^				SEM ^4^	*p*-Value
	0.00	5.24	10.48	15.72		
	Treatments ^3^					
	CTWB-0	CTWB-20	CTWB-40	CTWB-60		
**Observed dietary net energy**						
NE_m_, Mcal/kg DM	0.98	0.96	0.91	0.87	0.03	0.146
NE_g_, Mcal/kg DM	0.45	0.43	0.39	0.36	0.03	0.146
Expected DMI, kg DM/d	1.08 ^c^	1.26 ^b^	1.34 ^b^	1.50 ^a^	0.03	<0.001
**Observed-to-expected ratios**						
O:E DMI	1.88 ^a^	1.72 ^ab^	1.61 ^b^	1.47 ^b^	0.07	0.003
O:E NE_m_	0.575 ^c^	0.626 ^bc^	0.662 ^ab^	0.725 ^a^	0.02	0.002
O:E NE_g_	0.409 ^b^	0.460 ^ab^	0.490 ^ab^	0.563 ^a^	0.03	0.045
Observed DMI − expected DMI, kg DM/d	0.95	0.90	0.81	0.69	0.08	0.141
**Ultrasound measurements**						
FAT, mm	2.22 ^a^	2.06 ^ab^	1.92 ^b^	1.81 ^b^	0.08	0.004
LMA, cm^2^	11.42	11.12	10.35	10.49	0.37	0.157

^1^ NE_m_ = net energy for maintenance; NE_g_ = net energy for gain; DM = dry matter; DMI = dry matter intake; O:E = observed-to-expected ratio; FAT = rib fat thickness; LMA = longissimus muscle area. ^2^ Calculated from the analyzed condensed tannin (CT) concentration of CTWB and its dietary inclusion rate; CT in corn stover (CS) was not determined. ^3^ CTWB-0, CTWB-20, CTWB-40, and CTWB-60 correspond to diets containing 0, 20, 40, and 60% CTWB, respectively, representing progressive replacement of CS by CTWB. ^4^ Standard error of the mean. Values are presented as least squares means. ^a–c^ Means within a row with different superscripts differ (*p* ≤ 0.05) according to Tukey’s multiple comparison test. Higher O:E NE_m_ and NE_g_ ratios reflect lower calculated dietary energy concentrations used as reference values rather than greater energy utilization.

**Table 6:** Scrotal development and semen characteristics of Rambouillet ram lambs selected as breeding prospects and fed diets containing increasing levels of condensed tannin-containing woody biomass (CTWB; *n* = 36; 9 lambs/treatment).

Item	Condensed Tannins from CTWB, g/kg DM ^1^				SEM ^3^	*p*-Value
	0.00	5.24	10.48	15.72		
	Treatments ^2^					
	CTWB-0	CTWB-20	CTWB-40	CTWB-60		
Initial scrotal circumference, cm	27.70	27.71	27.67	27.54	0.57	0.996
Final scrotal circumference ^4^, cm	31.19	30.83	30.16	29.35	0.59	0.167
Change in scrotal circumference ^4^, cm	3.54	3.17	2.51	1.69	0.59	0.167
Sperm viability ^5^, %	49.30 ^b^	87.48 ^a^	81.06 ^ab^	78.26 ^ab^	5.80	0.032
Morphologically abnormal sperm ^5^, %	45.78 ^a^	13.32 ^b^	23.56 ^b^	5.78 ^b^	3.98	0.005
Total motility ^6^, %	82.54	76.44	83.54	93.62	5.15	0.161
Progressive motility ^6^, %	52.02	42.25	62.54	66.54	10.30	0.372
Non-progressive motility ^6^, %	30.01	32.76	21.40	33.96	5.81	0.408
Immotile sperm ^6^, %	17.42	23.67	16.57	7.67	5.20	0.240

^1^ Calculated from the analyzed condensed tannin (CT) concentration of CTWB and its dietary inclusion rate; CT in corn stover (CS) was not determined. ^2^ CTWB-0, CTWB-20, CTWB-40, and CTWB-60 correspond to diets containing 0, 20, 40, and 60% CTWB, respectively, representing the progressive replacement of corn stover by CTWB. ^3^ Values are presented as least squares means; SEM = standard error of the mean. Percentage outcomes were also analyzed after arcsine square-root transformation. Because this transformation did not alter the statistical conclusions, least squares means and SEM are presented on the original scale. ^4^ Final scrotal circumference and change in scrotal circumference were adjusted using initial scrotal circumference as a covariate. ^5^ Sperm viability and morphologically abnormal sperm were determined from independent counts of 50 spermatozoa per sample and expressed as percentages. Final values were adjusted using the corresponding baseline measurement as a covariate. ^6^ Computer-assisted sperm analysis variables correspond to the final evaluation and were adjusted using the corresponding baseline measurement as a covariate. Total motility comprised progressive and non-progressive motility. ^a,b^ Means within a row with different superscripts differ (*p* ≤ 0.05) according to Tukey’s multiple comparison test.

**Table 7:** Economic evaluation of Rambouillet ram lambs selected as breeding prospects and fed diets containing increasing levels of condensed tannin-containing woody biomass (CTWB; *n* = 36; 9 lambs/treatment).

**Item**	**Condensed Tannins from CTWB, g/kg DM ^1^**				**SEM ^3^**	***p*** **-Value**
	**0.00**	**5.24**	**10.48**	**15.72**		
	**Treatments ^2^**					
	**CTWB-0**	**CTWB-20**	**CTWB-40**	**CTWB-60**		
**Growth performance**						
Days on feed	70	70	70	70	-	-
Cumulative body weight change, kg	11.57	14.19	10.13	8.93	-	-
Dry matter intake, kg DM/d	2.00	2.15	2.18	2.17	-	-
**Feed costs**						
Diet cost ^4^, USD/kg DM	0.225	0.210	0.194	0.179	-	-
Total feed cost, USD/lamb	31.57	31.60	29.69	27.14	-	-
**Economic returns**						
Income from BW gain, USD/lamb	55.30	67.83	48.42	42.69	-	-
Income over feed cost ^5^, USD/lamb	23.74	36.20	18.68	15.33	7.29	0.217
Cost of gain ^5^, USD/kg BW gain	3.29	2.55	3.35	3.03	0.57	0.735

^1^ Calculated from the analyzed condensed tannin (CT) concentration of CTWB and its dietary inclusion rate; CT in corn stover (CS) was not determined. ^2^ CTWB-0, CTWB-20, CTWB-40, and CTWB-60 correspond to diets containing 0, 20, 40, and 60% condensed tannin-containing woody biomass (CTWB), respectively, representing progressive replacement of CS by CTWB. ^3^ When applicable, values are presented as least squares means from the final evaluation; SEM = standard error of the mean. A dash (-) indicates that no inferential statistical comparison was performed for that variable. ^4^ Diet costs were calculated from ingredient prices on a dry matter (DM) basis, and income from body weight (BW) gain was estimated using a live BW price of 4.78 USD/kg. ^5^ Income over feed cost (IOFC) and cost of gain (COG) were calculated for each individual lamb and analyzed using a one-way ANOVA including all treatment levels simultaneously. Treatment means were compared using Tukey’s multiple comparison test. Therefore, treatment means for IOFC and COG may not exactly equal simple arithmetic differences or ratios calculated from the rounded descriptive values shown in the table.

## Data Availability

The data presented in this study are available from the corresponding author upon reasonable request.

## Supplementary Materials

The following supporting information can be downloaded at: https://www.mdpi.com/article/10.3390/ani16203161/s1. Table S1: Scrotal development and semen characteristics across sampling days in Rambouillet ram lambs selected as breeding prospects and fed diets containing increasing levels of condensed tannin-containing woody biomass (CTWB; *n* = 36; 9 lambs/treatment).

## References

[1] Hellin J., Erenstein O., Beuchelt T., Camacho C., Flores D. (2013). Maize stover use and sustainable crop production in mixed crop–livestock systems in Mexico. Field Crops Res..

[2] Hernández-Briano P., Domínguez J.A., Rivera-Villegas A., Sánchez-Barbosa O.Y., Plascencia A., Rodríguez-Cordero D., Martínez-Guerrero O., Lazalde-Cruz R., Carrillo-Muro O. (2026). Inclusion of calcium propionate in a protein supplement for replacement beef heifers fed low-quality forage: Growth performance, body fat reserves, and economic return. Appl. Anim. Sci..

[3] Cooke A.S., Machekano H., Gwiriri L.C., Tinsley J.H.I., Silva G.M., Nyamukondiwa C., Safalaoh A., Morgan E.R., Lee M.R.F. (2025). The nutritional feed gap: Seasonal variations in ruminant nutrition and knowledge gaps in relation to food security in Southern Africa. Food Secur..

[4] Fourie P.J., Schwalbach L.M., Neser F.W.C., Van der Westhuizen C. (2004). Scrotal, testicular and semen characteristics of young Dorper rams managed under intensive and extensive conditions. Small Rumin. Res..

[5] National Research Council (2007). Nutrient Requirements of Small Ruminants: Sheep, Goats, Cervids, and New World Camelids.

[6] Maquivar M.G., Smith S.M., Busboom J.R. (2021). Reproductive management of rams and ram lambs during the pre-breeding season in US sheep farms. Animals.

[7] Castro M., Teixeira A., Fernández-Núñez E. (2021). The nutritive value of different Mediterranean browse species used as animal feeds under oak silvopastoral systems in Northern Portugal. Agrofor. Syst..

[8] Barry T.N., McNabb W.C. (1999). The implications of condensed tannins on the nutritive value of temperate forages fed to ruminants. Br. J. Nutr..

[9] Min B.R., Barry T.N., Attwood G.T., McNabb W.C. (2003). The effect of condensed tannins on the nutrition and health of ruminants fed fresh temperate forages: A review. Anim. Feed Sci. Technol..

[10] Zhao J., Jin Y., Du M., Liu W., Ren Y., Zhang C., Zhang J. (2017). The effect of dietary grape pomace supplementation on epididymal sperm quality and testicular antioxidant ability in ram lambs. Theriogenology.

[11] Li W., Yao R., Xie L., Liu J., Weng X., Yue X., Li F. (2021). Dietary supplementation of grape seed tannin extract stimulated testis development, changed fatty acid profiles and increased testis antioxidant capacity in pre-puberty Hu lambs. Theriogenology.

[12] Frutos P., Hervás G., Giráldez F.J., Mantecón A.R. (2004). Review: Tannins and ruminant nutrition. Span. J. Agric. Res..

[13] Nixon K.C., Kappelle M. (2006). Global and Neotropical distribution and diversity of oak (genus *Quercus*) and oak forests. Ecology and Conservation of Neotropical Montane Oak Forests.

[14] Paterson R.T. (1993). Use of Trees by Livestock 5: Quercus.

[15] Wilson A.D., Weir W.C., Torell D.T. (1971). Evaluation of chamise (*Adenostoma fasciculatum*) and interior live oak (*Quercus wislizenii*) as feed for sheep. J. Anim. Sci..

[16] Valencia Avalos S. (2004). Diversidad del género *Quercus* (Fagaceae) en México. Bol. Soc. Bot. Méx..

[17] Carrillo-Muro O., Ramírez-Lozano R.G., Castro-Rosales A.S., Hernández-Briano P., López-Carlos M.A., González-Ronquillo M., Rivera-Villegas A., Méndez-Llorente F., Aguilera-Soto J.I. (2018). Influence of landscape and collection period on yield, nutritive value and in vitro digestibility of *Quercus rugosa* leaf litter and its potential use as sheep feed. Small Rumin. Res..

[18] Carrillo-Muro O., Ramírez-Lozano R.G., Hernández-Briano P., López-Carlos M.A., Guerrero-Cervantes M., Rivera-Villegas A., Méndez-Llorente F., Aguilera-Soto J.I. (2020). Chemical composition, in vitro and in situ dry matter digestibility and preference of *Quercus resinosa* foliage. Indian J. Anim. Res..

[19] (1995). Especificaciones y Características Zoosanitarias Para el Transporte de Animales, Sus Productos y Subproductos, Productos Químicos, Farmacéuticos, Biológicos y Alimenticios Para Uso en Animales o Consumo Por Éstos.

[20] (1998). Trato Humanitario en la Movilización de Animales.

[21] (2001). Especificaciones Técnicas Para la Producción, Cuidado y Uso de Los Animales de Laboratorio.

[22] Association of Official Analytical Chemists (1995). Official Methods of Analysis.

[23] AOCS (2005). Official Method Am 5-04. Rapid Determination of Oil/Fat Utilizing High Temperature Solvent Extraction.

[24] Van Soest P.J., Robertson J.B., Lewis B.A. (1991). Methods for dietary fiber, neutral detergent fiber, and nonstarch polysaccharides in relation to animal nutrition. J. Dairy Sci..

[25] Porter L.J., Hrstich L.N., Chan B.G. (1986). The conversion of procyanidins and prodelphinidins to cyanidin and delphinidin. Phytochemistry.

[26] Theodorou M.K., Williams B.A., Dhanoa M.S., McAllan A.B., France J. (1994). A simple gas production method using a pressure transducer to determine the fermentation kinetics of ruminant feeds. Anim. Feed Sci. Technol..

[27] Menke K.H., Steingass H. (1988). Estimation of the energetic feed value obtained from chemical analysis and in vitro gas production using rumen fluid. Anim. Res. Dev..

[28] National Research Council (1984). Nutrient Requirements of Beef Cattle.

[29] Abdulrazak S.A., Nyangaga J., Fujihara T. (2001). Relative palatability to sheep of some browse species, their in sacco degradability and in vitro gas production characteristics. Asian-Australas. J. Anim. Sci..

[30] Ganguli A.C., Hale M.B., Launchbaugh K.L. (2010). Seasonal change in nutrient composition of spotted knapweed and preference by sheep. Small Rumin. Res..

[31] Launchbaugh K.L., Hodgson J., Illius A.W. (1996). Biochemical aspects of grazing behavior. The Ecology and Management of Grazing Systems.

[32] Brown D., Ng’ambi J.W., Norris D. (2016). Voluntary intake and palatability indices of Pedi goats fed different levels of *Acacia karroo* leaf meal by cafeteria method. Indian J. Anim. Res..

[33] Zinn R.A., Barreras A., Owens F.N., Plascencia A. (2008). Performance by feedlot steers and heifers: Daily gain, mature body weight, dry matter intake, and dietary energetics. J. Anim. Sci..

[34] Fuenmayor C., Martínez de Acurero M., Valle A., Fuentes A., Quintana H., Regueiro C. (1990). Observaciones sobre el desarrollo testicular y desprendimiento del prepucio en corderos West African, Barbados y Persa Cabeza Negra. Zootec. Trop..

[35] Baláži A., Vašíček J., Svoradová A., Macháč M., Jurčík R., Huba J., Pavlík I., Chrenek P. (2020). Comparison of three different methods for the analysis of ram sperm concentration. Slovak J. Anim. Sci..

[36] Peter A.T., Brito L., Althouse G., Aurich C., Chenoweth P., Fraser N., Lopate C., Love C., Luvoni G., Waberski D. (2021). Andrology laboratory review: Evaluation of sperm motility. Clin. Theriogenol..

[37] Palacin-Martinez C., Alvarez M., Montes-Garrido R., Neila-Montero M., Anel-Lopez L., de Paz P., Anel L., Riesco M.F. (2022). Frequency of semen collection affects ram sperm cryoresistance. Animals.

[38] SAS Institute Inc (2025). SAS OnDemand for Academics: SAS Studio.

[39] Priolo A., Waghorn G.C., Lanza M., Biondi L., Pennisi P. (2000). Polyethylene glycol as a means for reducing the impact of condensed tannins in carob pulp: Effects on lamb growth performance and meat quality. J. Anim. Sci..

[40] Vasta V., Pennisi P., Lanza M., Barbagallo D., Bella M., Priolo A. (2007). Intramuscular fatty acid composition of lambs given a tanniniferous diet with or without polyethylene glycol supplementation. Meat Sci..

[41] Bonanno A., Di Miceli G., Di Grigoli A., Frenda A.S., Tornambè G., Giambalvo D., Amato G. (2011). Effects of feeding green forage of sulla (*Hedysarum coronarium* L.) on lamb growth and carcass and meat quality. Animal.

[42] Valenti B., Natalello A., Vasta V., Campidonico L., Roscini V., Mattioli S., Pauselli M., Priolo A., Lanza M., Luciano G. (2019). Effect of different dietary tannin extracts on lamb growth performances and meat oxidative stability: Comparison between mimosa, chestnut and tara. Animal.

[43] Hervás G., Frutos P., Ramos G., Giráldez F.J., Mantecón A.R. (2004). Intraruminal administration of two doses of quebracho tannins to sheep: Effect on rumen degradation and total tract digestibility, faecal recovery and toxicity. J. Anim. Feed Sci..

[44] Rojas-Román L.A., Castro-Pérez B.I., Estrada-Angulo A., Angulo-Montoya C., Yocupicio-Rocha J.A., López-Soto M.A., Barreras A., Zinn R.A., Plascencia A. (2017). Influence of long-term supplementation of tannins on growth performance, dietary net energy and carcass characteristics: Finishing lambs. Small Rumin. Res..

[45] Rivera-Méndez C.R., Plascencia A., Torrentera N., Zinn R.A. (2016). Influence of tannins supplementation on growth performance, dietary net energy and carcass characteristics of yearling steers fed finishing diet containing dried distillers grains with solubles. Indian J. Anim. Sci..

[46] Keddam R., Bouderoua K., El-Affifi M., Selselet-Attou G. (2010). Growth performances, carcasses parameters and meat fatty acid composition of lamb fed green oak acorns (*Quercus ilex*) based diet. Afr. J. Biotechnol..

[47] Kheradmand A., Babaei H., Batavani R.A. (2006). Effect of improved diet on semen quality and scrotal circumference in the ram. Vet. Arh..

[48] Bester N., Schwalbach L.M.J., van der Merwe H.J., Greyling J.P.C., Fair M.D. (2004). The influence of dietary energy concentrations on scrotal, testicular and semen characteristics of young Dorper rams. S. Afr. J. Anim. Sci..

