# Supplementary material for: Effects of Progressive Replacement of Corn Stover with Condensed Tannin-Containing Woody Biomass on Growth Performance and Reproductive Development of Prospective Breeding Ram Lambs
Source: Animals (Basel). 2026 Oct 9;16(20):3161. doi: 10.3390/ani16203161 (PMC13700685; doi:10.3390/ani16203161)
Supplement: Supplementary file 1 [file animals-16-03161-s001.zip › animals-4598712-supplementary.pdf]

Supplementary Materials

Effects of Progressive Replacement of Corn Stover with Condensed Tannin-Containing Woody Biomass on Growth Performance and Reproductive Development of Prospective Breeding Ram Lambs

Table S1. Scrotal development and semen characteristics across sampling days in Rambouillet ram lambs selected as breeding prospects and fed diets containing increasing levels of condensed tannin-containing woody biomass (CTWB; 36 lambs assigned; 9 lambs/treatment).

Item	Day	Condensed tannins from CTWB, g/kg DM [1]				SEM [3]	*p*-value
		0.00	5.24	10.48	15.72		
		Treatments [2]					
		CTWB-0	CTWB-20	CTWB-40	CTWB-60		
Scrotal circumference, cm	14	28.11	27.95	27.93	26.87	0.77	0.653
	28	29.20	28.00	27.58	27.99	0.62	0.351
	42	28.48	28.14	27.49	27.38	0.64	0.592
	56	31.58	30.14	29.66	28.72	0.81	0.144
	70	31.19	30.83	30.16	29.35	0.59	0.167
Total motility[4], %	14	80.38	88.24	84.42	82.79	6.09	0.813
	28	76.94	81.18	83.61	77.13	7.58	0.921
	42	76.41	80.24	88.33	89.55	4.71	0.186
	56	90.21	85.42	92.10	74.17	6.36	0.253
	70	82.54	76.44	83.54	93.62	5.15	0.161
Progressive motility[4], %	14	52.78	57.21	47.23	54.70	9.02	0.858
	28	62.84	62.99	47.73	50.29	9.20	0.503
	42	44.07	52.16	64.88	53.35	8.66	0.441
	56	67.06	60.76	67.99	44.01	8.37	0.215
	70	52.02	42.25	62.54	66.54	10.30	0.372
Non-progressive motility[4], %	14	30.71	34.53	32.60	20.75	6.21	0.440
	28	15.49[b]	20.17[b]	34.66[a]	23.49[ab]	3.74	0.014
	42	32.85	29.04	21.98	37.08	6.45	0.436
	56	24.53	26.98	24.04	28.20	5.29	0.935
	70	30.01	32.76	21.40	33.96	5.81	0.408
Immotile sperm[4], %	14	19.69	11.53	17.42	15.81	6.35	0.805
	28	22.81	18.44	16.57	23.56	7.63	0.914
	42	23.19	14.26	11.18	11.98	4.30	0.231
	56	9.87	14.44	8.31	25.53	6.45	0.300
	70	17.42	23.67	16.57	7.67	5.20	0.240
Sperm viability[5], %	14	66.02	78.26	67.46	72.10	7.50	0.621
	28	89.78	78.16	75.02	77.96	3.91	0.263
	42	78.80	60.96	81.20	71.04	17.78	0.812

	56	97.90^{a}	85.56^{a}	58.62^{b}	80.76^{ab}	5.43	0.031
	70	49.30^{b}	87.48^{a}	81.06^{ab}	78.26^{ab}	5.80	0.032
Morphologically abnormal sperm[5], %	14	27.90^{ab}	21.30^{ab}	34.36^{a}	17.86^{b}	2.65	0.028
	28	8.96	17.98	19.98	19.40	2.33	0.077
	42	12.74	30.62	15.50	21.80	15.64	0.818
	56	6.76	18.04	39.32	22.54	3.23	0.073
	70	45.78^{a}	13.32^{b}	23.56^{b}	5.78^{b}	3.98	0.005

[1] Calculated from the analyzed condensed tannin (CT) concentration of CTWB and its dietary inclusion rate; CT in corn stover (CS) was not determined. [2] CTWB-0, CTWB-20, CTWB-40, and CTWB-60 correspond to diets containing 0, 20, 40, and 60% condensed tannin-containing woody biomass, respectively, representing the progressive replacement of corn stover by CTWB. [3] Values are presented as least squares means; SEM = standard error of the mean. Each post-baseline evaluation was analyzed by ANCOVA using the corresponding d 0 measurement as a covariate. Percentage outcomes were also analyzed after arcsine square-root transformation. Because the transformation did not alter the statistical conclusions, least squares means and SEM are presented on the original scale. [4] Computer-assisted sperm analysis variables were adjusted using the corresponding baseline measurement as a covariate. Total motility comprised progressive and non-progressive motility. [5] Sperm viability and morphologically abnormal sperm were determined from independent counts of 50 spermatozoa per sample and expressed as percentages. [a],[b] Means within a row with different superscripts differ ($p \leq 0.05$) according to Tukey's multiple comparison test.
